# Inflammation-Based Prognostic Scores in Pancreatic Cancer Patients—A Single-Center Analysis of 1294 Patients within the Last Decade

**DOI:** 10.3390/cancers15082367

**Published:** 2023-04-19

**Authors:** Christopher C. M. Neumann, François Schneider, Georg Hilfenhaus, Loredana Vecchione, Matthäus Felsenstein, Jana Ihlow, Dominik Geisel, Steffen Sander, Johann Pratschke, Sebastian Stintzing, Ulrich Keilholz, Uwe Pelzer

**Affiliations:** 1 Department of Hematology, Oncology and Tumor Immunology, Charité-Universitätsmedizin Berlin, Freie Universität Berlin, Humboldt-Universität zu Berlin, and Berlin Institute of Health, 10117 Berlin, Germany; 2 Department of Surgery CCM/CVK, Charité-Universitätsmedizin Berlin, Freie Universität Berlin, Humboldt-Universität zu Berlin, and Berlin Institute of Health, 10117 Berlin, Germany; 3 Department of Pathology, Charité-Universitätsmedizin Berlin, Freie Universität Berlin, Humboldt-Universität zu Berlin, and Berlin Institute of Health, 10117 Berlin, Germany; 4 Department of Radiology, Charité-Universitätsmedizin Berlin, Freie Universität Berlin, Humboldt-Universität zu Berlin, and Berlin Institute of Health, 10117 Berlin, Germany; 5 Charité Comprehensive Cancer Center, Charité-Universitätsmedizin Berlin, 10117 Berlin, Germany

**Keywords:** pancreatic cancer, inflammatory biomarkers, neutrophil to lymphocyte ratio (NLR), platelet to lymphocyte ratio (PLR), lymphocyte to monocyte ratio (LMR), CRP to albumin ratio (CAR), inflammatory benchmark index (IBI)

## Abstract

**Simple Summary:**

Inflammation markers such as the neutrophil to lymphocyte ratio (NRL), the platelet to lymphocyte ratio (PLR), the lymphocyte to monocyte ratio (LMR) and the CRP to albumin ratio (CAR) have recently gained attention due to their potential as prognostic scores for overall survival (OS) in pancreatic cancer. So far, these parameters have not been validated in a larger cohort to predict OS in terms of potential recurrence after curative intended resection or in terms of patient response to chemotherapy. In the largest single-center analysis of 1294 patients, inflammation markers were compared and a new highly significant combined score, termed the inflammatory benchmark index (IBI), comprising five inflammatory markers was developed. In multivariate analyses NLR (*p* = 0.001), LMR (*p* = 0.038), CAR (*p* < 0.001) and IBI (*p* < 0.001) were identified as independent prognostic markers for overall survival.

**Abstract:**

Inflammatory properties are known to promote tumor progression leading to an impaired median overall survival (mOS). Various small studies have focused on a wide range of inflammation-based prognostic indicators. By using sufficient data from 1294 out of 2323 patients diagnosed with pancreatic cancer between 2009 and 2021 at our cancer center, inflammatory markers such as the neutrophil to lymphocyte ratio (NRL), the platelet to lymphocyte ratio (PLR), the lymphocyte to monocyte ratio (LMR) and the CRP to albumin ratio (CAR) were evaluated. We identified a new combined score, termed the inflammatory benchmark index (IBI). We performed univariate and multivariate overall survival analyses and identified optimal prognostic cut-off values for each parameter. In univariate analyses, advanced age (*p* < 0.001), gender (*p* < 0.001), tumor stage (*p* < 0.001), CA19-9 (*p* = 0.001), NLR (*p* = 0.001), LMR (*p* = 0.004), PLR (*p* = 0.004), CAR (*p* = 0.001) and IBI (*p* = 0.001) were identified as prognostic markers. In multivariate analyses advanced age (*p* < 0.001), gender (*p* = 0.001), tumor stage (*p* < 0.001), CA19-9 (*p* < 0.001), NLR (*p* = 0.001), LMR (*p* = 0.038), CAR (*p* < 0.001) and IBI (*p* < 0.001) were independent prognostic markers. These findings emphasize the impact of inflammation in pancreatic cancer, provide easily accessible prognostic values for the clinician, and may be useful as stratification parameters for trials aimed at patient inflammation or immune response.

## 1. Introduction

Pancreatic cancer represents one of the most aggressive types of cancer with a very poor prognosis. With only 10–20% of all patients presenting at an operable stage at initial diagnosis, surgical resection remains the only curative treatment option with a 5-year survival rate of 30% after resection [1]. Because of its high heterogeneity and insufficient understanding of therapeutical effects, there still is an urgent need for prognostic biomarkers.

Inflammation represents one of the key factors of tumor carcinogenesis. Based on histological analyses, a correlation of the magnitude of immune cell infiltration to the clinical outcome has been observed for patients with pancreatic cancer [2]. Moreover, there has been growing evidence for the predictive value of inflammatory biomarkers in regard to overall survival (OS) of cancer patients. Parameters reflecting chronic inflammation are the neutrophil to lymphocyte ratio (NLR), the platelet to lymphocyte ratio (PLR), as well as the lymphocyte to monocyte ratio (LMR) [3,4,5]. Another newly developed parameter is the CRP to albumin ratio (CAR) taking into account the inflammatory as well as the nutritional status [6].

By current clinical guidelines borderline-resectable tumors are defined by anatomic, biological and conditional factors [7]. When the serum carbohydrate antigen (CA) 19-9 is higher than 500 units/mL tumors are classified as biologically not resectable with curative intention. However, inflammatory biomarkers have not yet been taken into account for this group of patients.

In order for inflammation-based prognostic scores to be integrated into future clinical practice, we conducted a retrospective analysis of a large patient cohort (N = 1294) of resectable, locally advanced and metastasized pancreatic cancer patients in which we correlated the NLR, PLR, LMR and CAR to the median overall survival (mOS). Moreover, we developed a new combined score called the inflammatory benchmark index (IBI), proving to be highly significant in uni- as well as multivariate analyses independent of tumor stage and of ECOG status.

## 2. Materials and Methods

We conducted a retrospective analysis by screening 2323 patients diagnosed and documented with pancreatic cancer at the Charité-Universitätsmedizin Berlin between 2009 and 2021. This study was approved by the Charité ethics committee (EA/071/22). A clear question of the project was defined: Do inflammatory markers at the point of diagnosis entail prognostic value in pancreatic cancer? Data were included and excluded based on clearly defined criteria based on the PICOTS structure (population, index prognostic factor, comparator prognostic factors, outcome, timing, setting) [8]. The data were extracted from the hospital’s patient records strictly following the CHARMS-PF (checklist for critical appraisal and data extraction for systemic reviews of prediction modelling studies) [9]. The design of this study was in accordance with the recommended prognostic guidelines published by Riley et al. [8]. The research questions were addressed within the PROGRESS (PROGnosis RESearch Strategy) Framework. First, we defined the health outcome of patients with pancreatic cancer to be the overall survival. Second, we identified known and novel inflammatory scores as prognostic factors for overall survival. Third, we developed, validated and examined the effect of known and novel inflammatory scores for the prediction of overall survival. In total, 2323 patients with a confirmed diagnosis of pancreatic cancer were screened for available differential blood count documentation in our database. Patients without reported differential blood count values at initial diagnosis and/or unclear metastatic status were excluded. Based on these criteria, a total of 1294 patients were selected for further analysis (see Supplementary Material, Figure S1). Patients were allocated to three groups: resected (n = 537), non-resected/non-metastasized (n = 134) and metastasized (n = 623). In order for the reported differential blood count to be included in analyses, a maximum duration of four weeks after the point of verified diagnosis was tolerated. Moreover, the measurement of the differential blood count had to be prior to any kind of treatment intervention such as surgery and chemotherapy.

NLR, PLR, LMR, CAR and IBI were calculated based on the reported differential blood count values. The NLR was calculated by dividing the neutrophil blood count (/nL) by the lymphocyte count (/nL). The PLR and LMR were calculated in analogy to the NLR. The CAR was calculated by dividing CRP (mg/L) by serum albumin (g/dL).

The inflammatory benchmark index (IBI) was calculated as follows:

$$IBI=\frac{Lymphocytes(/nL)+Monocytes(/nL)+Neutrophils(/nL)+Platelets(/nL)}{CRP(mg/L)}$$

In order for new inflammatory scores to be developed, we screened all possible combinations with three or four parameters of the differential blood count (neutrophils, lymphocytes, monocytes, platelets). For each potential score an individual cut-off value was determined and the mOS, as well as its statistical significance in univariate as well as multivariate analysis, was assessed. To exclude any influence of repeated blood measurements in multivariate analyses, one inflammation-based parameter was included in the analysis at time.

The primary endpoint was set on mOS by the duration from the date of the verified diagnosis to the date of the last follow-up or death. We conducted uni- and multivariate analyses to identify correlations of inflammation-based parameters with respect to mOS.

To determine optimized cut-off values for each parameter, we performed time-dependent receiver operating characteristic (ROC) curves based on the Youden method.

We calculated Kaplan–Meier plots to determine the mOS for the NLR, PLR, LMR, CAR and IBI. We compared survival curves for each group using Kaplan–Meier methodology and the log-rank test. Statistical analysis was conducted for the entire patient cohort as well as for subgroups of resected, locally advanced and metastasized patients (see Supplementary Figures S2–S5). As ECOG is known to be a strong prognostic factor for survival in pancreatic cancer [10], we also conducted subgroup analyses of the individual parameters NLR, PLR, LMR, CAR and IBI by excluding patients with unknown ECOG status.

Statistical significance was defined as *p* < 0.05. Results of Cox regression modeling are presented as hazard ratios (HR) and associated 95% confidence interval (CI). In addition, we reviewed NLR cut-off values that had already been presented in previously published studies.

## 3. Results

We enrolled 1294 out of 2323 patients with a confirmed diagnosis of pancreatic cancer treated at the Charité-Universitätsmedizin Berlin between 2009 and 2021. For all of these patients, a differential blood count report prior to any kind of intervention (surgery, chemotherapy) was documented (see Table S1).

### 3.1. Patient Characteristics

In the selected cohort of 1294 patients with pancreatic cancer, the median age was 66 years, ranging from 28 to 94 years. Of these, 576 patients (45.0%) were female and 718 (55.0%) were male. ECOG status was mostly ≤2 (616 patients, 47.6%) with the vast majority of performance states not being sufficiently documented (486, 37.6%). A total of 537 patients (41.5%) were resected, whilst 134 patients (10.5%) were non-resected/non-metastasized and 623 (48.0%) were metastasized. In most cases, the tumor was localized in the head of the pancreas (675 patients, 52.2%). In 125 cases (9.7%) the tumor was located in the body, in 208 (16.1%) in the tail and in 286 (22.0%) of the cases the location was either overlapping or unknown. Out of the 537 resected patients, the pathological resection margin was R0 in 347 (27%), R1 in 147 (11%) and R2 in 4 (0.3%) of all cases. Patients who were not resected in curative intention received palliative treatment with the recommendation of systemic chemotherapy and/or best-supportive care (Table 1).

### 3.2. Determination of Cut-Off Values

To determine optimized cut-off values for each parameter, we performed time-dependent receiver operating characteristic (ROC) curves based on the Youden method. For the individual parameters of the differential blood count and the inflammatory marker, CRP, the corresponding cut-off value for neutrophils was 5/nL, lymphocytes 1.35/nL, monocytes 0.6/nL, platelets 235/nL and CRP 15 mg/L. We identified the NLR cut-off value at 4.0. Cut-off values for the LMR, PLR, CAR and IBI were 1.6, 180, 0.4 and 30, respectively.

### 3.3. Predictive Factors of Survival

Following identification of optimal cut-off values, we correlated individual inflammatory parameters by univariate analyses with respect to the median overall survival (mOS; Table 2). An mOS of 15 vs. 9 months was observed for neutrophils below 5/nL. Longer mOS was also observed for reported values of lymphocytes above 1.35 (12 vs. 9 months), platelets above 235/nL (11 vs. 9 months), albumin above 38.5 g/L (15 vs. 8 months), monocytes below 0.60/nL (13 vs. 9 months) and CRP below 15/nL (15 vs. 6 months). Moreover, a better mOS was assessed for the following ratios including IBI: NLR below 4.0 (14 vs. 8 months), LMR above 1.6 (12 vs. 7 months), PLR below 180 (12 vs. 9 months), CAR below 4.0 (16 vs. 6 months) and an IBI above 30 (16 vs. 7 months). In the subgroup analyses of resected, locally advanced and metastasized patients, the NLR and CAR proved to be statistically significant in univariate analyses for all tumor stages (see Supplementary Figures S2–S5). For the PLR and LMR, the inflammatory scores remained statistically significant.

**Figure 1:**
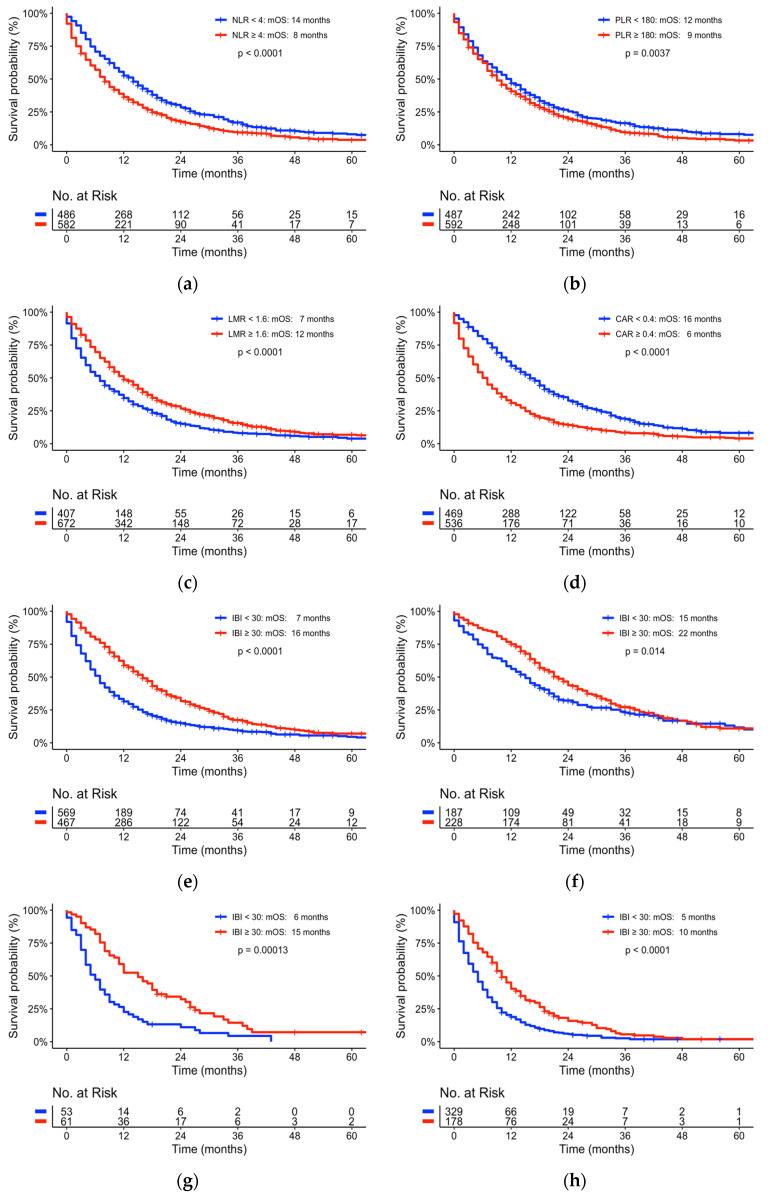
Kaplan–Meyer curve univariate comparison of median overall survival representing cut-off values of: (**a**) NLR = 4; (**b**) PLR = 1.6; (**c**) LMR = 180; (**d**) CAR = 0.4; (**e**) IBI = 30 for entire patient cohort; (**f**) IBI = 30 for resected patient cohort; (**g**) IBI = 30 for locally advanced patient cohort; (**h**) IBI = 30 for metastasized patient cohort. NLR—neutrophil to lymphocyte ratio; PLR—platelet to lymphocyte ratio; LMR—lymphocyte to monocyte ratio; CAR—CRP to albumin ratio; IBI—inflammatory benchmark index; mOS—median overall survival (for further subgroup analyses see Supplementary Material, Tables S2–S5).

As ECOG is a strong and robust prognostic factor for overall survival [10], we conducted a further subgroup analysis excluding patients with unknown ECOG status. We evaluated Kaplan–Meier curves of the total patient population for NLR, LMR, PLR, CAR and IBI (Supplementary Figures S6 and S7 and Figure 1e–h). For lower ECOG status (0–1) the NLR, CAR and IBI were identified to be prognostic scores for overall survival independent of ECOG status. NLR, LMR, PLR, CAR and IBI were all independent prognostic scores for overall survival of patients with higher ECOG status (see Supplementary Figures S6 and S7).

### 3.4. Assessing New Predictive Inflammatory Scores of Survival

Having screened variations of all possible combinations with three or four parameters of the differential blood count (neutrophils, lymphocytes, monocytes, platelets (see Supplementary Tables S5–S7)), a total of 24 different scores (A to X) were evaluated. Out of the 24 scores, 16 proved to be significant in the univariate and multivariate analyses. Compared to all 24 different scores, the IBI remained the statistically significant score with the largest difference in median overall survival below and above the determined cut-off value of 30.

### 3.5. Univariate and Multivariate Analyses of Patient Cohort Characteristics and Inflammatory-Based Markers

For identification of prognostic factors affecting the overall survival of pancreatic cancer patients, univariate and multivariate analyses were performed (Table 3). In the univariate analysis, advanced age (*p* < 0.001), gender (*p* < 0.001), tumor stage (*p* < 0.001), CA19-9 (*p* = 0.001), NLR (*p* < 0.001), LMR (*p* = 0.004), PLR (*p* = 0.004), CAR (*p* = 0.001) and IBI (*p* = 0.001) were identified as prognostic markers. In the multivariate analyses advanced age (*p* < 0.001), gender (*p* = 0.001), tumor stage (*p* < 0.001), CA19-9 (*p* < 0.001), NLR (*p* = 0.001), LMR (*p* = 0.038), CAR (*p* < 0.001) and IBI (*p* = 0.001) represented independent prognostic markers. The pancreatic localization (head, body, tail) of the tumor was not affecting the overall survival of patients in our cohort study; though for the majority of cases reported data of tumor localization was unavailable.

The mOS was significantly longer for a lower NLR (*p* < 0.001, see Figure 1a), lower PLR (*p* = 0.004, see Figure 1c) and lower CAR (*p* = 0.001, see Figure 1d), as well as a higher LMR (*p* = 0.004, see Figure 1b) and higher IBI (*p* = 0.001).

## 4. Discussion

By assessing inflammatory-based prognostic markers, we were able to demonstrate statistical correlations of advanced age (*p* < 0.001), gender (*p* = 0.001), tumor stage (*p* < 0.001), CA19-9 (*p* < 0.001), NLR (*p* = 0.001), LMR (*p* = 0.038) and CAR (*p* < 0.001) to overall survival in multivariate analyses, thus emphasizing their role as independent prognostic biomarkers in pancreatic cancer. To our knowledge, this retrospective study represents the largest patient cohort of 1294 patients at a single high-volume cancer center and thus represents robust data. Whilst the range of other retrospective studies accounted for cohorts between 54 and a maximum of 474 patients (see Table 4), the patient cohort included in this study accounted for a sample size similar to formally published meta-analyses.

As previously reported by Hanahan et al., tumor-promoting inflammation represents one of the hallmarks of cancer, representing a key role in tumorigenesis, malignant transformation, tumor progression and metastasis [11]. While inflammation caused by hepatitis B, human papillomavirus and Helicobacter pylori is known to promote cancer development, a malignant tumor itself can trigger regional inflammatory reactions. This in turn leads to a release of pro-inflammatory cytokines such as interleukin-1,-2,-6, VEGF, TNF-α and TGF-β [12].

The induced inflammatory reaction within the microenvironment of malignancies causes a systemic elevation of neutrophils, platelets and C-reactive protein (CRP), as well as a depletion of lymphocytes. The exact mechanisms of these changes, however, have not yet been fully elucidated. By tumor secretion of IL-6, for example, megakaryocytes are promoted, resulting in elevated platelet levels [13]. Moreover, it has been reported that tumor secretion of TNF-α and IL-10 leads to lymphopenia and lymphocyte dysfunction [14,15]. As lymphocyte infiltration correlates with long-term oncological outcomes in pancreatic cancer, lymphopenia presents a means of immune evasion [2].

Based on these findings, many cohort studies analyzed the effect on immune cells and their corresponding inflammatory biomarkers on the overall survival of pancreatic cancer patients. In our study cohort, we were able to correlate high levels of lymphocytes and thrombocytes as well as low levels of neutrophils, monocytes and leukocytes to a longer mOS (see Table 2).

The most commonly reported inflammatory parameter is the NLR. Low NLR values are known to correlate with longer median overall survival in various retrospective study cohorts (see Table 4). Up to present, the largest meta-analysis considering the NLR included 40,599 patients from 57 cohort studies including various types of solid tumors (breast, renal, HCC, ovarian, etc.). The NLR was found to be highly significant (HR = 1.81, 95% CI = 1.67–1.97, *p* < 0.001) with respect to mOS. Moreover, a meta-analysis of 8252 patients including 43 cohort studies of pancreatic cancer patients also underscored the relevance of the NLR as an independent prognostic marker for mOS (HR = 1.81, 95% CI = 1.59–2.05, *p* < 0.00001) [16]. In agreement with previous findings, the NLR of our study cohort proved to be an independent prognostic marker in the multivariate analysis. Whereas other study cohorts only considered certain subgroups of pancreatic cancer patients, specifically resected-only or metastasized-only, this study cohort comprises the entire spectrum of patients, empowering the multivariate prognostic impact. Moreover, it should be emphasized that no clear cut-off values of the NLR have been agreed on. When examining our study cohort, we identified the optimal cut-off value to be 4.0, which is in the range of and in very good agreement with previously reported values varying from 2 to 5 [16]. While the majority of NLR studies reported a statistical significance, some studies with smaller patient cohorts did not find a correlation between mOS and NLR [17,18,19], indicating the vast heterogeneity of former study cohorts.

The LMR is another inflammatory-based prognostic marker that has been examined over the last years (see Table 4). A meta-analysis by Hu et al. was able to include 10 cohort studies with 2557 patients suffering from pancreatic cancer. A low LMR was correlated to a shorter mOS (HR = 0.60, 95% CI = 0.50–0.71, *p* < 0.001) with cut-off values varying from 2.05 to 4.62 [20]. When assessing our study population, the LMR was identified to be an independent prognostic marker with a specific cut-off value of 1.6. The optimal cut-off value (1.6) determined with ROC analysis for our study cohort, however, was lower than the range of previously reported studies. As former LMR studies were based on smaller cohorts of 97 to a maximum of 474 patients, one could argue that the calculated cut-off value of our cohort with 1294 patients provides a more robust assessment. Additionally, some studies pointed out the LMR to be not statistically significant with respect to mOS [3,21].

Another inflammatory-based marker to be considered is represented by the PLR. In a meta-analysis of 17 cohorts a low PLR was linked to longer mOS (HR = 1.28, 95% CI = 1.17–1.40, *p* = 0.00001). Cut-off values ranged from 126 to 300 [22]. Many retrospective study cohorts identified the PLR to be an independent multivariate factor for mOS (see Table 4). Others did not identify a correlation of PLR with mOS [17,18]. In our study cohort, the PLR was the only inflammatory marker which did not show a statistical multivariate correlation to the overall survival.

As ECOG is a strong and robust prognostic factor for overall survival [10], further subgroup analyses of our cohort proved the NLR, CAR and the IBI to be independent of ECOG status.

Of course, there are limitations of the study presented. First, a retrospective cohort was used. Second, the database only includes patients treated at a single high-volume center. Independent validation is necessary to integrate the score or the results obtained into clinical practice. A weakening limitation for our determined score is that we are currently not able to validate the results with an independent cohort. The available data from other working groups are only available for smaller patient numbers, with which no meaningful validation according to given guidelines is possible. The further project plan is to validate the data on the entirety of the patients in our further prospective single-center cohort with the data of cooperating working groups to have a sufficient validation cohort.

Third, the current study did not take into account other hallmarks of cancer besides inflammation and did not stratify by pre-existing illness, genetic/mutational status of the tumor, therapeutic regimens and tumor stroma content. Moreover, in addition to peripheral immune cell counts there are also genetic inflammatory markers accessible. Genes such as deoxyribonuclease 1-like 3 (DNASE1L3), encoding for deoxyribonuclease gamma, have previously been identified as additional inflammation-based markers [23]. A DNASE1L3 deficiency, for example, results in chronic inflammation with respect to anti-DNA responses and autoimmune diseases such as systemic lupus erythematosus [24]. Higher expressions of DNASE1L3 were recently correlated to longer overall survival after radical resection of hepatocellular carcinoma [24].

For the purpose of the largest single-center retrospective study presented here, however, one may assume that certain variabilities might be compensated for.

**Table 4:** Summary of reported retrospective analyses of inflammation-based prognostic indicators in pancreatic cancer.

Author	Year	Country	Patient No	Parameter	Cut-Offs	Multivariate HR (95% CI)	Status
Sierzega [25]	2017	Poland	54	NLR	5.0	1.66 [1.12–2.46]; *p* = 0.012	Resected
Pointer [3]	2020	USA	277	NLR	5.0	2.13 [1.41–3.22]; *p* = 0.002	Resected
Giakoustidis [26]	2018	UK	127	NLR	4.0	2.05 [1.11–3.78]; *p* = 0.020	Resected
Iawi [27]	2020	Japan	119	NLR	3.7	2.43 [1.48–3.98]; *p* < 0.001	Non-Resectable
Ventriglia [4]	2018	Italy	70	NLR	5.0	2.77 [1.30–5.70]; *p* = 0.006	Metastasized
McLellan [5]	2020	France	172	NLR	5.0	2.01 [1.33–3.05]; *p* = 0.001	Metastasized
Giakoustidis [26]	2018	UK	127	PLR	120.0	1.47 [0.88–2.45]; *p* = 0.138	Resected
Martin [28]	2015	Australia	124	PLR	200.0	1.158 [1.07–2.33]; *p* = 0.020	Non-Resectable
Li [29]	2019	China	134	PLR	123.0	1.72 [1.16–2.55]; *p* = 0.007	Metastasized
Sierzega [25]	2017	Poland	54	LMR	3.0	1.65 [1.06–2.58]; *p* = 0.026	Resected
Li [30]	2016	China	144	LMR	2.6	0.15 [0.09–0.25]; *p* < 0.001	Resected
Stotz [31]	2015	Austria	474	LMR	2.8	0.81 [0.66–0.99]; *p* = 0.040	Mixed
Xue [32]	2017	Japan	405	LMR	2.8	0.46 [0.31–0.69]; *p* < 0.001	Non-Resectable + Metastasized
Van Wijk [33]	2020	Netherlands	163	CAR	0.20	1.75 [1.20–2.54]; *p* = 0.004	Resected
Funamizu [34]	2022	Japan	203	CAR	0.09	34.51 [11.75–101.38]; *p* < 0.001	Resected
Haruki [35]	2016	Japan	113	CAR	0.03	1.73 [1.04–2.87]; *p* = 0.035	Resected
Fan [36]	2019	China	595	CAR	0.18	1.84 [1.51–2.24]; *p* < 0.001	Non-Resectable + Metastasized

## 5. Conclusions

In conclusion, we were able to present robust data on the largest retrospective study cohort of a single high-volume center analyzing inflammatory-based prognostic markers. We identified the NLR, LMR and CAR to be independent predictive biomarkers with respect to the median overall survival of patients suffering from pancreatic cancer. We were also able to identify and confirm optimal cut-off values for our study population. These values were in accordance with previously reported cut-off values. Moreover, we systematically screened all possible combinations of potential inflammatory scores and identified an optimal combinational score termed the inflammatory benchmark index (IBI). This newly developed score includes not only two values of the differential blood count, but all four values (neutrophils, lymphocytes, monocytes, platelets) and CRP. The score fits both the adjuvant and palliative situation (Figure 1e–h), thus reflecting the basic biological character of the cancer disease. In addition to tumor stage, this easily accessible prognostic benchmark index may provide additive guidance for further treatment decision-making and may be useful as a stratification parameter for trials aimed at patient inflammation or immune response.

## Figures and Tables

**Table 1:** Descriptive statistics of study cohort.

Factor	Total No. (%)
No. of patients Median Age (range)	1294 66 (28–94) years
Sex	
Female	576 (45.0)
Male	718 (55.0)
ECOG	
≥2	192 (14.8)
<2 Unknown	616 (47.6) 486 (37.6)
Stage	
Resected	537 (41.5)
Locally advanced	134 (10.5)
Metastasized	623 (48.0)
Localization	
Head	675 (52.2)
Body	125 (9.7)
Tail	208 (16.1)
Overlap	63 (4.9)
Not specified	223 (17.1)
Treatment	
Curative~	
R0	347 (26.7)
R1	147 (11.5)
R2	4 (0.3)
RX	39 (3.0)
Palliative~	757 (58.5)

**Table 2:** Correlation of individual parameters with median overall survival.

Factor	Cut-Off	mOS (Months) ≥Cut-Off	mOS (Months) <Cut-Off	*p*-Value
Neutrophils (/nL)	5	9	15	0.001
Lymphocytes (/nL)	1.35	12	9	0.001
Monocytes (/nL)	0.60	9	13	0.001
Platelets (/nL)	235	11	9	0.190
CRP (mg/L)	15	6	15	0.001
Albumin (g/L)	38.5	15	8	<0.0001
NLR	4.0	8	14	0.0001
LMR	1.6	12	7	<0.0001
PLR	180	9	12	0.0037
CAR	0.4	6	16	<0.0001
IBI	30	16	7	0.0001

mOS—median overall survival; CRP—C-reactive protein; NLR—neutrophil to lymphocyte ratio, PLR—platelet to lymphocyte ratio; LMR—lymphocyte to monocyte ratio; CAR—CRP to albumin ratio; IBI—inflammatory benchmark index.

**Table 3:** Univariate and multivariate logistic regression analyses of study cohort.

Factor	Univariate Analysis				Multivariate Analysis		
	N	HR	95% CI	*p*-Value	HR	95% CI	*p*-Value
Age (years)							
≥65	594						
<65	700	0.75	0.70–0.85	<0.001	0.8	0.7–0.9	<0.001
Sex							
Female	576						
Male	718	1.2	1.1–1.4	<0.001	1.4	1.1–1.5	0.001
Tumor stage							
Resected	537						
Locally advanced	134	1.8	1.4–2.2	<0.001	1.8	1.2–1.5	<0.001
Metastasized	623	2.6	2.2–3.0	<0.001	2.5	2.1–2.9	<0.001
Localization							
Head	675						
Body	125	0.95	0.76–1.2	0.618	0.86	0.6–1.1	0.249
Tail	208	1.20	0.99–1.4	0.058	0.98	0.8–1.2	0.862
Overlap	63	1.49	1.12–2.0	0.007	1.01	0.7–1.4	0.970
Not specified	223	1.50	1.26–1.8	0.001	1.11	0.9–1.3	0.295
CA19-9 (kU/L)							
≥300	611						
<300	524	0.55	0.48–0.64	0.001	1.5	1.2–1.7	<0.001
Unknown CA19-9	159						
NLR							
<4	581						
≥4	713	1.5	1.2–1.6	0.001	1.3	1.1–1.7	0.0011
LMR							
<1.6	491						
≥1.6	803	0.69	0.61–0.79	0.001	0.8	0.7–0.99	0.0383
PLR							
<180	590						
≥180	704	1.2	1.1–1.4	0.004	1.0	0.9–1.2	0.625
CAR							
<0.4	561						
≥0.4	636	1.8	1.61–2.1	0.001	1.4	1.2–1.7	<0.001
Unknown CRP	97						
IBI							
<30	689						
≥30	552	0.57	0.51–0.65	0.001	0.65	0.6–0.8	<0.001
Unknown IBI	53						

NLR—neutrophil to lymphocyte ratio; PLR—platelet to lymphocyte ratio; LMR—lymphocyte to monocyte ratio; CAR—CRP to albumin ratio; IBI—inflammatory benchmark index; CRP—C-reactive protein.

## Data Availability

The database is stored on the Charité’s own server in a legally secure manner. All data were saved and checked in pseudonymized form. The pseudonymized data set can be requested from the project manager of the investigation (UP), if there is contractual legal protection.

## Supplementary Materials

The following supporting information can be downloaded at: https://www.mdpi.com/article/10.3390/cancers15082367/s1, Figure S1: Study flow chart; Figure S2: Kaplan–Meyer curve univariate comparison of subgroup analyses of NLR; Figure S3: Kaplan–Meyer curve univariate comparison of subgroup analyses of PLR; Figure S4: Kaplan-Meier curve univariate comparison of subgroup analyses of LMR; Figure S5: Kaplan-Meier curve univariate comparison of subgroup analyses of CAR; Figure S6: Kaplan-Meier curve univariate comparison of subgroup analyses for ECOG status 0–1; Figure S7: Kaplan-Meier curve univariate comparison of subgroup analyses for ECOG status ≥ 2; Table S1: Descriptive statistics of study cohort with respect to NLR; Table S2: Descriptive statistics of study cohort with respect to PLR; Table S3: Descriptive statistics of study cohort with respect to LMR; Table S4: Descriptive statistics of study cohort with respect to CAR; Table S5: Descriptive statistics of study cohort with respect to IBI; Table S6: Different inflammation-based parameters; Table S7: Univariate and multivariate logistic regression analyses scores from Table S6.

## References

[1] Strobel O., Neoptolemos J., Jäger D., Büchler M.W. (2019). Optimizing the Outcomes of Pancreatic Cancer Surgery. Nat. Rev. Clin. Oncol..

[2] Orhan A., Vogelsang R.P., Andersen M.B., Madsen M.T., Hölmich E.R., Raskov H., Gögenur I. (2019). The Prognostic Value of Tumour-Infiltrating Lymphocytes (TILs) in Pancreatic Cancer: A Systematic Review and Meta-Analysis. Ann. Oncol..

[3] Pointer D.T., Roife D., Powers B.D., Murimwa G., Elessawy S., Thompson Z.J., Schell M.J., Hodul P.J., Pimiento J.M., Fleming J.B. (2020). Neutrophil to Lymphocyte Ratio, Not Platelet to Lymphocyte or Lymphocyte to Monocyte Ratio, Is Predictive of Patient Survival after Resection of Early-Stage Pancreatic Ductal Adenocarcinoma. BMC Cancer.

[4] Ventriglia J., Petrillo A., Huerta Alváro M., Laterza M.M., Savastano B., Gambardella V., Tirino G., Pompella L., Diana A., Iovino F. (2018). Neutrophil to Lymphocyte Ratio as a Predictor of Poor Prognosis in Metastatic Pancreatic Cancer Patients Treated with Nab-Paclitaxel plus Gemcitabine: A Propensity Score Analysis. Gastroenterol. Res. Pract..

[5] McLellan P., Henriques J., Ksontini F., Doat S., Hammel P., Desrame J., Trouilloud I., Louvet C., Pietrasz D., Vernerey D. (2021). Prognostic Value of the Early Change in Neutrophil-to-Lymphocyte Ratio in Metastatic Pancreatic Adenocarcinoma. Clin. Res. Hepatol. Gastroenterol..

[6] Zang Y., Fan Y., Gao Z. (2020). Pretreatment C-Reactive Protein/Albumin Ratio for Predicting Overall Survival in Pancreatic Cancer: A Meta-Analysis. Medicine.

[7] Isaji S., Mizuno S., Windsor J.A., Bassi C., Fernández-Del Castillo C., Hackert T., Hayasaki A., Katz M.H.G., Kim S.-W., Kishiwada M. (2018). International Consensus on Definition and Criteria of Borderline Resectable Pancreatic Ductal Adenocarcinoma 2017. Pancreatol. Off. J. Int. Assoc. Pancreatol. IAP Al.

[8] Riley R.D., Moons K.G.M., Snell K.I.E., Ensor J., Hooft L., Altman D.G., Hayden J., Collins G.S., Debray T.P.A. (2019). A Guide to Systematic Review and Meta-Analysis of Prognostic Factor Studies. BMJ.

[9] Moons K.G.M., de Groot J.A.H., Bouwmeester W., Vergouwe Y., Mallett S., Altman D.G., Reitsma J.B., Collins G.S. (2014). Critical Appraisal and Data Extraction for Systematic Reviews of Prediction Modelling Studies: The CHARMS Checklist. PLoS Med..

[10] Colloca G. (2022). Performance Status as Prognostic Factor in Phase III Trials of First-Line Chemotherapy of Unresectable or Metastatic Pancreatic Cancer: A Trial-Level Meta-Analysis. Asia Pac. J. Clin. Oncol..

[11] Hanahan D. (2022). Hallmarks of Cancer: New Dimensions. Cancer Discov..

[12] Padoan A., Plebani M., Basso D. (2019). Inflammation and Pancreatic Cancer: Focus on Metabolism, Cytokines, and Immunity. Int. J. Mol. Sci..

[13] Alexandrakis M.G., Passam F.H., Moschandrea I.A., Christophoridou A.V., Pappa C.A., Coulocheri S.A., Kyriakou D.S. (2003). Levels of Serum Cytokines and Acute Phase Proteins in Patients With Essential and Cancer-Related Thrombocytosis. Am. J. Clin. Oncol..

[14] Bellone G., Turletti A., Artusio E., Mareschi K., Carbone A., Tibaudi D., Robecchi A., Emanuelli G., Rodeck U. (1999). Tumor-Associated Transforming Growth Factor-Beta and Interleukin-10 Contribute to a Systemic Th2 Immune Phenotype in Pancreatic Carcinoma Patients. Am. J. Pathol..

[15] Salazar-Onfray F., López M.N., Mendoza-Naranjo A. (2007). Paradoxical Effects of Cytokines in Tumor Immune Surveillance and Tumor Immune Escape. Cytokine Growth Factor Rev..

[16] Zhou Y., Wei Q., Fan J., Cheng S., Ding W., Hua Z. (2018). Prognostic Role of the Neutrophil-to-Lymphocyte Ratio in Pancreatic Cancer: A Meta-Analysis Containing 8252 Patients. Clin. Chim. Acta.

[17] Chawla A., Huang T.L., Ibrahim A.M., Hardacre J.M., Siegel C., Ammori J.B. (2018). Pretherapy Neutrophil to Lymphocyte Ratio and Platelet to Lymphocyte Ratio Do Not Predict Survival in Resectable Pancreatic Cancer. HPB.

[18] Jamieson N.B., Denley S.M., Logue J., MacKenzie D.J., Foulis A.K., Dickson E.J., Imrie C.W., Carter R., McKay C.J., McMillan D.C. (2011). A Prospective Comparison of the Prognostic Value of Tumor- and Patient-Related Factors in Patients Undergoing Potentially Curative Surgery for Pancreatic Ductal Adenocarcinoma. Ann. Surg. Oncol..

[19] Zhou W., Kuang T., Han X., Chen W., Xu X., Lou W., Wang D. (2020). Prognostic Role of Lymphocyte-to-Monocyte Ratio in Pancreatic Neuroendocrine Neoplasms. Endocr. Connect..

[20] Hu R.-J., Ma J.-Y., Hu G. (2018). Lymphocyte-to-Monocyte Ratio in Pancreatic Cancer: Prognostic Significance and Meta-Analysis. Clin. Chim. Acta Int. J. Clin. Chem..

[21] Abe T., Nakata K., Kibe S., Mori Y., Miyasaka Y., Ohuchida K., Ohtsuka T., Oda Y., Nakamura M. (2018). Prognostic Value of Preoperative Nutritional and Immunological Factors in Patients with Pancreatic Ductal Adenocarcinoma. Ann. Surg. Oncol..

[22] Zhou Y., Cheng S., Fathy A.H., Qian H., Zhao Y. (2018). Prognostic Value of Platelet-to-Lymphocyte Ratio in Pancreatic Cancer: A Comprehensive Meta-Analysis of 17 Cohort Studies. OncoTargets Ther..

[23] Xiao Y., Yang K., Liu P., Ma D., Lei P., Liu Q. (2022). Deoxyribonuclease 1-like 3 Inhibits Hepatocellular Carcinoma Progression by Inducing Apoptosis and Reprogramming Glucose Metabolism. Int. J. Biol. Sci..

[24] Wang S., Ma H., Li X., Mo X., Zhang H., Yang L., Deng Y., Yan Y., Yang G., Liu X. (2020). DNASE1L3 as an Indicator of Favorable Survival in Hepatocellular Carcinoma Patients Following Resection. Aging.

[25] Sierzega M., Lenart M., Rutkowska M., Surman M., Mytar B., Matyja A., Siedlar M., Kulig J. (2017). Preoperative Neutrophil-Lymphocyte and Lymphocyte-Monocyte Ratios Reflect Immune Cell Population Rearrangement in Resectable Pancreatic Cancer. Ann. Surg. Oncol..

[26] Giakoustidis A., Neofytou K., Costa Neves M., Giakoustidis D., Louri E., Cunningham D., Mudan S. (2018). Identifying the Role of Neutrophil-to-Lymphocyte Ratio and Platelets-to-Lymphocyte Ratio as Prognostic Markers in Patients Undergoing Resection of Pancreatic Ductal Adenocarcinoma. Ann. Hepato-Biliary-Pancreat. Surg..

[27] Iwai N., Okuda T., Sakagami J., Harada T., Ohara T., Taniguchi M., Sakai H., Oka K., Hara T., Tsuji T. (2020). Neutrophil to Lymphocyte Ratio Predicts Prognosis in Unresectable Pancreatic Cancer. Sci. Rep..

[28] Martin H.L., Ohara K., Kiberu A., Van Hagen T., Davidson A., Khattak M.A. (2014). Prognostic Value of Systemic Inflammation-Based Markers in Advanced Pancreatic Cancer. Intern. Med. J..

[29] Li W., Chen Y., Wang X., Shi Y., Dai G., Li X. (2019). Pretreatment Platelet to Lymphocyte Ratio Is Predictive of Overall Survival in Metastatic Pancreatic Ductal Adenocarcinoma. Transl. Cancer Res..

[30] Li G.-J., Xu H.-W., Ji J.-J., Yang F., Gao B.-Q. (2016). Prognostic Value of Preoperative Lymphocyte-to-Monocyte Ratio in Pancreatic Adenocarcinoma. OncoTargets Ther..

[31] Stotz M., Szkandera J., Stojakovic T., Seidel J., Samonigg H., Kornprat P., Schaberl-Moser R., Seggewies F., Hoefler G., Gerger A. (2015). The Lymphocyte to Monocyte Ratio in Peripheral Blood Represents a Novel Prognostic Marker in Patients with Pancreatic Cancer. Clin. Chem. Lab. Med..

[32] Xue P., Hang J., Huang W., Li S., Li N., Kodama Y., Matsumoto S., Takaori K., Zhu L., Kanai M. (2017). Validation of Lymphocyte-to-Monocyte Ratio as a Prognostic Factor in Advanced Pancreatic Cancer: An East Asian Cohort Study of 2 Countries. Pancreas.

[33] van Wijk L., de Klein G.W., Kanters M.A., Patijn G.A., Klaase J.M. (2020). The Ultimate Preoperative C-Reactive Protein-to-Albumin Ratio Is a Prognostic Factor for Survival after Pancreatic Cancer Resection. Eur. J. Med. Res..

[34] Funamizu N., Utsunomiya T., Honjo M., Ito C., Shine M., Uraoka M., Nagaoka T., Tamura K., Sakamoto K., Ogawa K. (2022). Preoperative C-Reactive Protein-to-Albumin Ratio Predicts Postoperative Pancreatic Fistula Following Pancreatoduodenectomy: A Single-Center, Retrospective Study. Curr. Oncol..

[35] Haruki K., Shiba H., Shirai Y., Horiuchi T., Iwase R., Fujiwara Y., Furukawa K., Misawa T., Yanaga K. (2016). The C-Reactive Protein to Albumin Ratio Predicts Long-Term Outcomes in Patients with Pancreatic Cancer After Pancreatic Resection. World J. Surg..

[36] Fan Z., Fan K., Gong Y., Huang Q., Yang C., Cheng H., Jin K., Ni Q., Yu X., Luo G. (2019). The CRP/Albumin Ratio Predicts Survival And Monitors Chemotherapeutic Effectiveness In Patients With Advanced Pancreatic Cancer. Cancer Manag. Res..

