# Supplementary material for: Inflammation-Based Prognostic Scores in Pancreatic Cancer Patients—A Single-Center Analysis of 1294 Patients within the Last Decade
Source: Cancers (Basel). 2023 Apr 19;15(8):2367. doi: 10.3390/cancers15082367 (PMC10136644; doi:10.3390/cancers15082367)
Supplement: Supplementary file 1 [file cancers-15-02367-s001.zip › cancers-2296505-supplementary.pdf]

Supplementary Materials:

Figure S1. Study flow chart.

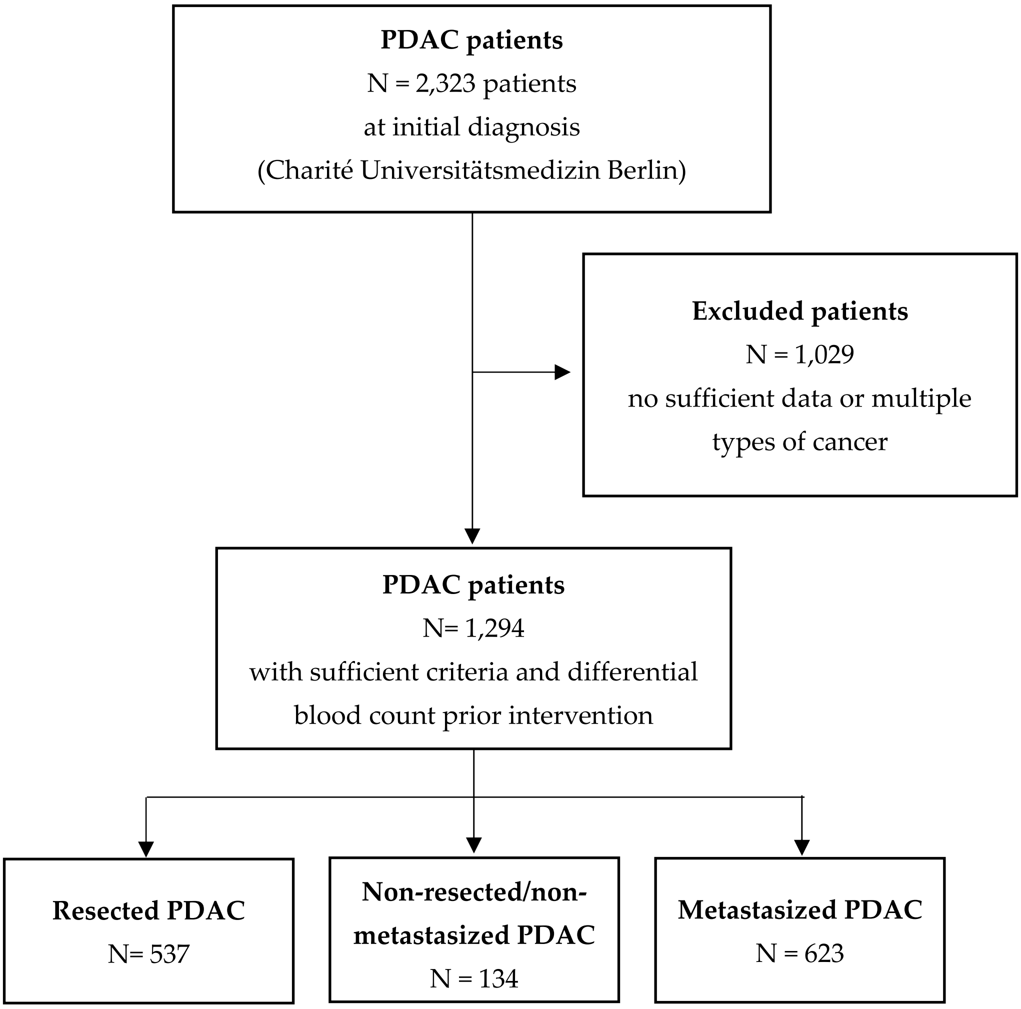

PDAC – pancreatic adenocarcinoma.

Table S1. Descriptive statistics of study cohort with respect to NLR.

Factor	Overall<br>N = 1,294	NLR < 4<br>N = 581	NLR ≥ 4<br>N = 713
**Age (years)**	66 (28– 94)	64 (28 – 87)	69 (34 – 94)
**Sex**			
Female	576 (44.5)	285 (22.0)	291 (22.5)
Male	718 (55.5)	298 (23.0)	420 (32.5)
**ECOG**			
≥ 2	192 (14.8)	83 (6.4)	109 (8.4)
< 2	616 (47.6)	296 (22.9)	320 (24.7)
Unknown	486 (37.6)	202 (15.6)	284 (22.0)
**Stage**			
Resected	537 (41.5)	228 (17.6)	309 (23.9)
Locally advanced	134 (10.5)	76 (6.0)	58 (4.5)
Metastasized	623 (48.0)	277 (21.3)	346 (26.7)
**Localization**			
Head	675 (52.2)	301 (23.2)	374 (29.0)
Body	125 (9.7)	69 (5.2)	56 (4.5)
Tail	208 (16.1)	85 (6.6)	123 (9.5)
Overlap	63 (4.9)	26 (2.0)	37 (2.9)
Not-specified	223 (17.1)	100 (7.6)	123 (9.5)
**Treatment**			
Resection			
R0	347 (26.7)	147 (11.2)	200 (15.5)
R1	147 (11.5)	63 (5.0)	84 (6.5)
R2	4 (0.3)	1 (0.1)	3 (0.2)
RX	5 (0.4)	3 (0.2)	2 (0.2)
Unknown	34 (2.6)	28 (2.2)	6 (0.4)
Palliative Treatment	757 (58.5)	339 (26.2)	418 (23.3)

NLR – neutrophile to lymphocyte ratio.

Table S2. Descriptive statistics of study cohort with respect to PLR.

Factor	Overall<br>N = 1,294	PLR < 180<br>N = 590	PLR ≥ 180<br>N = 704
**Age (years)**	66 (28– 94)	66 (28 – 87)	68 (34 – 94)
**Sex**			
Female	576 (44.5)	274 (21.1)	302 (23.3)
Male	718 (55.5)	316 (24.4)	402 (31.2)
**ECOG**			
≥ 2	192 (14.8)	76 (5.8)	116 (9.0)
< 2	616 (47.6)	294 (22.7)	322 (24.9)
Unknown	486 (37.6)	220 (17.1)	266 (20.5)
**Stage**			
Resected	537 (41.5)	235 (18.2)	302 (23.3)
Locally advanced	134 (10.5)	66 (5.1)	68 (5.4)
Metastasized	623 (48.0)	289 (22.3)	334 (25.7)
**Localization**			
Head	675 (52.2)	277 (21.4)	398 (30.8)
Body	125 (9.7)	79 (6.1)	46 (3.6)
Tail	208 (16.1)	100 (7.7)	108 (8.4)
Overlap	63 (4.9)	30 (2.3)	33 (2.5)
Not-specified	223 (17.1)	104 (8.0)	119 (9.1)
**Treatment**			
Resection			
R0	347 (26.7)	157 (12.1)	190 (14.6)
R1	147 (11.5)	62 (4.8)	85 (6.7)
R2	4 (0.3)	1 (0.1)	3 (0.2)
RX	5 (0.4)	3 (0.2)	2 (0.2)
Unknown	34 (2.6)	28 (2.2)	6 (0.4)
Palliative Treatment	757 (58.5)	339 (26.2)	418 (23.3)

PLR – platelet to lymphocyte ratio.

Table S3. Descriptive statistics of study cohort with respect to LMR.

Factor	Overall<br>N = 1,294	LMR < 1.6<br>N = 491	LMR ≥ 1.6<br>N = 803
**Age (years)**	66 (28– 94)	68 (34 – 94)	66 (28 – 87)
**Sex**			
Female	576 (44.5)	181 (14.0)	394 (30.5)
Male	718 (55.5)	308 (23.8)	410 (31.7)
**ECOG**			
≥ 2	192 (14.8)	83 (6.4)	109 (8.4)
< 2	616 (47.6)	218 (16.9)	398 (30.7)
Unknown	486 (37.6)	222 (17.1)	264 (20.5)
**Stage**			
Resected	537 (41.5)	209 (16.1)	328 (25.4)
Locally advanced	134 (10.5)	32 (2.4)	102 (8.0)
Metastasized	623 (48.1)	250 (19.2)	373 (28.9)
**Localization**			
Head	675 (52.2)	268 (20.7)	407 (31.5)
Body	125 (9.7)	44 (3.4)	81 (6.2)
Tail	208 (16.1)	70 (5.4)	138 (10.7)
Overlap	63 (4.9)	25 (1.9)	38 (3.0)
Not-specified	223 (17.2)	84 (6.5)	139 (10.7)
**Treatment**			
Resection			
R0	347 (26.7)	129 (9.9)	218 (16.8)
R1	147 (11.5)	61 (4.7)	86 (6.8)
R2	4 (0.3)	2 (0.15)	2 (0.15)
RX	5 (0.4)	1 (0.08)	4 (0.32)
Unknown	34 (2.6)	16 (1.2)	18 (1.4)
Palliative Treatment	757 (58.5)	282 (21.8)	475 (36.7)

LMR – lymphocyte to monocyte ratio.

Table S4. Descriptive statistics of study cohort with respect to CAR.

Factor	Overall N = 1,197 [1]	CAR < 0.4 N = 561	CAR ≥ 0.4 N = 636
**Age (years)**	66 (28– 94)	66 (28 – 88)	67 (35 – 94)
**Sex**			
Female	530 (44.3)	277 (23.1)	253 (21.2)
Male	667 (55.7)	284 (23.7)	383 (32.0)
**ECOG**			
≥ 2	181 (15.1)	54 (4.5)	127 (12.3)
< 2	568 (47.5)	282 (23.6)	286 (23.9)
Unknown	448 (37.4)	225 (18.8)	223 (18.7)
**Stage**			
Resected	515 (43.0)	285 (23.8)	230 (19.2)
Locally advanced	123 (10.3)	73 (6.1)	50 (4.2)
Metastasized	559 (46.7)	203 (17.0)	356 (29.8)
**Localization**			
Head	632 (52.8)	312 (26.1)	320 (26.8)
Body	112 (9.4)	63 (5.3)	49 (4.1)
Tail	192 (16.1)	72 (6.0)	120 (10.0)
Overlap	58 (4.9)	27 (2.3)	31 (2.6)
Not-specified	203 (17.0)	87 (7.3)	116 (9.7)
**Treatment**			
Resection			
R0	337 (28.1)	189 (15.8)	148 (12.3)
R1	141 (11.8)	78 (6.5)	63 (5.3)
R2	3 (0.3)	2 (0.2)	1 (0.1)
RX	5 (0.4)	3 (0.3)	2 (0.2)
Unknown	29 (2.4)	13 (1.1)	16 (1.3)
Palliative Treatment	682 (57.0)	276 (23.1)	406 (34.0)

CAR – CRP to albumin ratio; CRP – C-reactive protein; [1] in the case of 97 out of 1,294 patients no CRP value was recorded.

Table S5. Descriptive statistics of study cohort with respect to IBI.

Factor	Overall N = 1,241[1]	IBI < 30 N = 689	IBI ≥ 30 N = 552
**Age (years)**	66 (28– 94)	67 (35 – 94)	65 (28 – 87)
**Sex**			
Female	550 (44.3)	282 (22.7)	268 (21.6)
Male	691 (55.7)	407 (32.8)	284 (22.9)
**ECOG**			
≥ 2	187 (15.1)	130 (10.5)	57 (4.6)
< 2	586 (47.2)	321 (25.9)	265 (21.3)
Unknown	468 (37.7)	238 (19.2)	230 (18.5)
**Stage**			
Resected	520 (41.9)	239 (19.3)	281 (22.6)
Locally advanced	129 (10.4)	60 (4.8)	69 (5.6)
Metastasized	592 (47.7)	390 (31.4)	202 (16.3)
**Localization**			
Head	652 (52.5)	338 (27.2)	314 (25.3)
Body	115 (9.3)	54 (4.4)	61 (4.9)
Tail	199 (16.0)	130 (10.5)	69 (5.5)
Overlap	59 (4.8)	31 (2.5)	28 (2.3)
Not-specified	216 (17.4)	136 (10.9)	80 (6.5)
**Treatment**			
Resection			
R0	291 (23.4)	143 (11.5)	148 (11.9)
R1	135 (10.9)	64 (5.2)	71 (5.7)
R2	2 (0.2)	0 (0.0)	2 (0.2)
RX	13 (1.0)	9 (0.7)	4 (0.3)
Unknown	43 (3.5)	20 (1.6)	23 (1.9)
Palliative Treatment	757 (61.0)	453 (36.5)	304 (24.5)

IBI – Inflammatory Benchmark-Index. [1] in the case of 53 out of 1,294 patients no IBI value was recorded.

Table S6. Different combined scores using inflammation-based parameters.

Score	Ratios
**IBI**	$\frac{L+M+N+P}{CRP}$
**A**	$L+M+N+P$
**B**	$\frac{L+M+N}{P}$
**C**	$\frac{L+N+P}{M}$
**D**	$\frac{L+M+P}{N}$
**E**	$\frac{M+N+P}{L}$
**F**	$\frac{L+M}{N+P}$
**G**	$\frac{L+N}{M+P}$
**H**	$\frac{L+P}{M+N}$
**I**	$L+M+N$
**J**	$\frac{L+M}{N}$
**K**	$\frac{L+N}{M}$
**L**	$\frac{M+N}{L}$
**M**	$L+M+P$
**N**	$\frac{L+M}{P}$
**O**	$\frac{L+P}{N}$
**P**	$\frac{M+P}{N}$
**Q**	$L+N+P$
**R**	$\frac{L+N}{P}$
**S**	$\frac{L+P}{N}$
**T**	$\frac{N+P}{L}$
**U**	$M+N+P$
**V**	$\frac{M+N}{P}$
**W**	$\frac{M+P}{N}$
**X**	$\frac{N+P}{M}$

IBI – Inflammation-Benchmark-Index; L – Lymphocytes (/nl); M – Monocytes (/nl); N – neutrophiles (/nl); P – Platelets (/nl); CRP – C-reactive protein (mg/dl).

Table S7. Univariate and multivariate logistic regression analyses of combined scores.

Score	Cut-off	mOS (months) < Cut-off	mOS (months) ≥ Cut-off	Univariate analysis HR	Univariate analysis 95% CI	Univariate analysis P-value	Multivariate analysis HR	Multivariate analysis 95% CI	Multivariate analysis P-value
**IBI**	30	7	16	0.6	0.5 – 0.65	< 0.001	0.7	0.6 – 0.8	< 0.001
**A**	226	9	11	0.9	0.8 – 1.1	0.213	0.9	0.8 – 1.1	0.774
**B**	0.04	12	8	1.3	1.1 – 1.5	< 0.001	1.5	1.3 – 1.8	< 0.001
**C**	469	9	14	0.9	0.7 – 0.9	< 0.001	0.8	0.7 – 0.9	0.01
**D**	40	7	13	1.3	0.7 – 2.4	0.419	1.3	0.6 – 2.5	0.529
**E**	190	12	9	1.3	1.1 – 1.4	< 0.001	1.2	1.0 – 1.4	0.003
**F**	0.015	10	7	1.2	1.0 – 1.5	0.058	1.2	1.0 – 1.6	0.072
**G**	0.04	12	8	1.3	1.1 – 1.5	< 0.001	1.5	1.3 – 1.8	< 0.001
**H**	36	8	13	0.7	0.6 – 0.8	< 0.001	0.7	0.5 – 0.8	< 0.001
**I**	9	11	8	1.3	1.1 – 1.4	< 0.001	1.4	1.2 – 1.6	< 0.001
**J**	0.4	8	14	0.7	0.6 – 0.8	< 0.001	0.7	0.6 – 0.8	< 0.001
**K**	14	10	10	1.0	0.9 – 1.2	0.602	1.2	1.0 – 1.4	0.058
**L**	5	14	18	1.4	1.3 – 1.6	< 0.001	1.4	1.2 – 1.6	< 0.001
**M**	238	9	11	0.9	0.8 – 1.1	0.278	1.0	0.8 – 1.1	0.501
**N**	0.015	10	7	1.2	1 – 1.5	0.077	1.1	0.8 – 1.8	0.460
**O**	39	7	13	0.71	0.6 – 0.8	< 0.001	0.6	0.5 – 0.7	< 0.001
**P**	45	8	13	0.72	0.6 – 0.8	< 0.001	0.7	0.6 – 0.8	< 0.001
**Q**	244	9	11	0.94	0.8 – 1.1	0.336	1.0	0.8 – 1.1	0.719
**R**	0.035	12	8	1.3	1.1 – 1.5	< 0.001	1.4	1.2 – 1.7	< 0.001
**S**	39	7	13	0.7	0.6 – 0.8	< 0.001	0.6	0.5 – 0.7	< 0.001
**T**	189	12	9	1.2	1.1 – 1.4	< 0.001	1.2	1.1 – 1.4	< 0.001
**U**	224	9	11	0.9	0.8 – 1.1	0.273	1.0	0.8 – 1.1	0.862
**V**	0.025	13	8	1.3	1.2 – 1.5	< 0.001	1.4	1.2 – 1.6	< 0.001
**W**	45	8	13	0.7	0.6 – 0.8	< 0.001	0.7	0.6 – 0.8	< 0.001
**X**	464	9	14	0.8	0.7 – 0.9	< 0.001	0.8	0.7 – 0.9	< 0.001

IBI – Inflammation-Benchmark-Index; mOS – median Overall Survival.

Figure S2. Kaplan-Meyer Curves univariate comparison of sub-group analyses of NLR: (**a**) resected patients; (**b**) locally advanced; (**c**) metastasized patients.

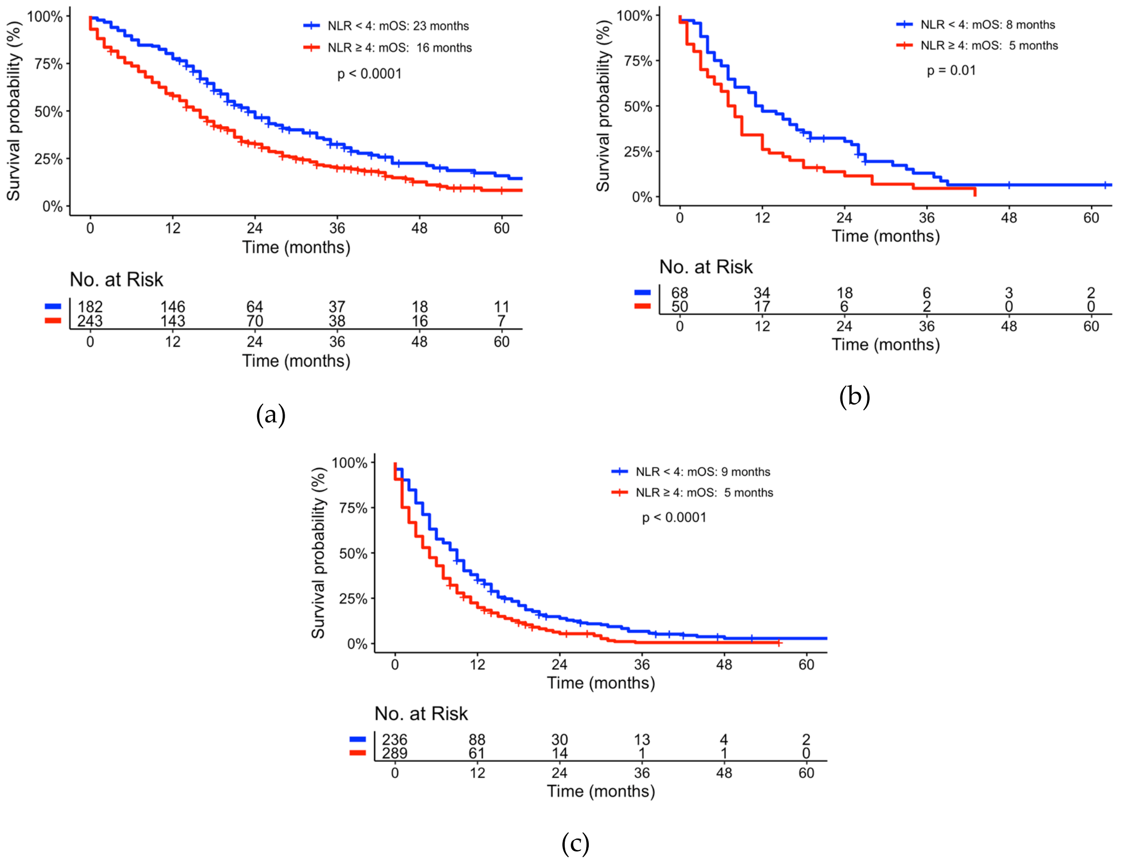

NLR - neutrophile to lymphocyte ratio.

Figure S3. Kaplan-Meyer Curves univariate comparison of sub-group analyses of PLR: (**a**) resected patients; (**b**) locally advanced; (**c**) metastasized patients.

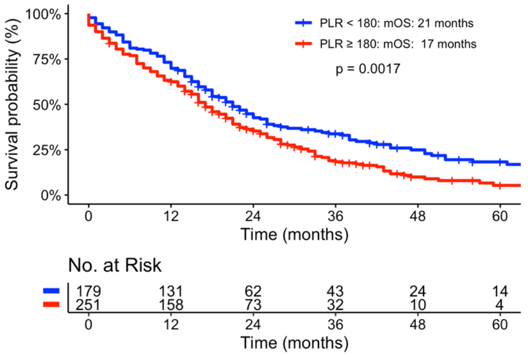

(a)

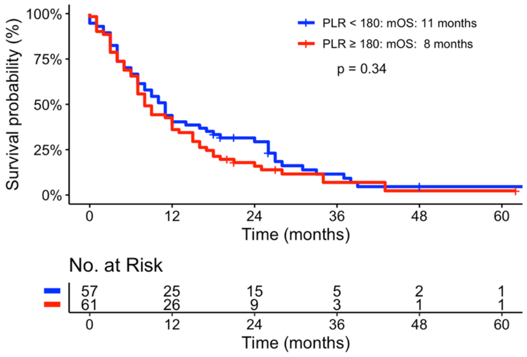

(b)

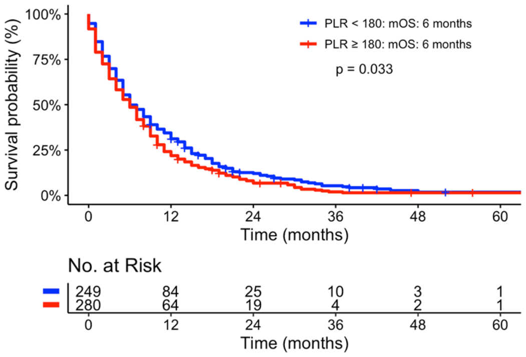

(c)

PLR - platelet to lymphocyte ratio.

Figure S4. Kaplan-Meyer Curves univariate comparison of sub-group analyses of LMR: (**a**) resected patients; (**b**) locally advanced; (**c**) metastasized patients.

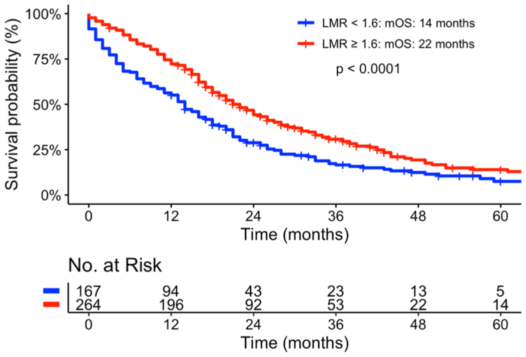

(a)

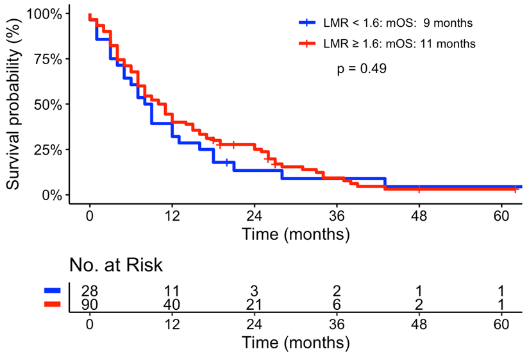

(b)

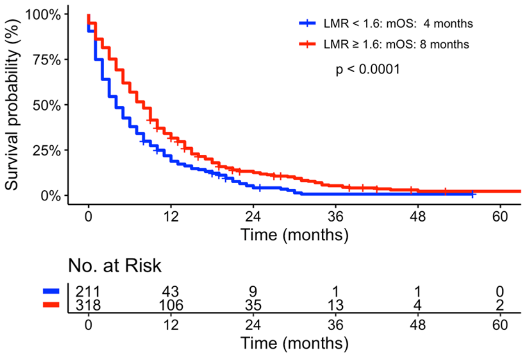

(c)

LMR - lymphocyte to monocyte ratio.

Figure S5. Kaplan-Meyer Curves univariate comparison of sub-group analyses of CAR: (**a**) resected patients; (**b**) locally advanced; (**c**) metastasized patients.

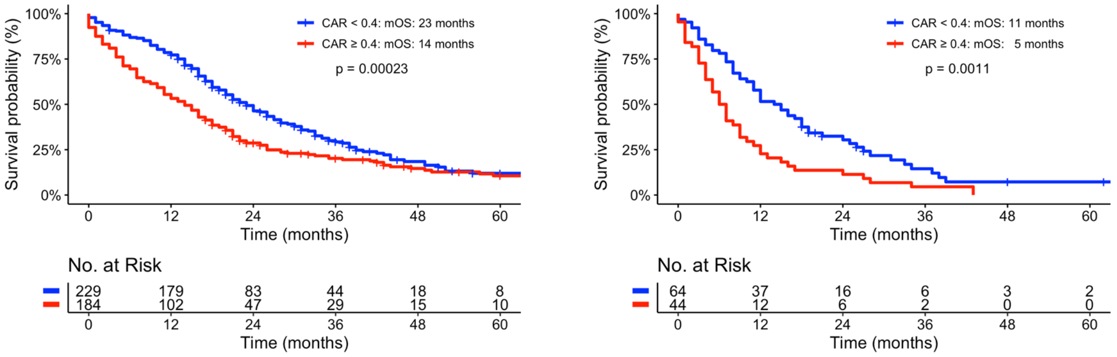

(a) (b)

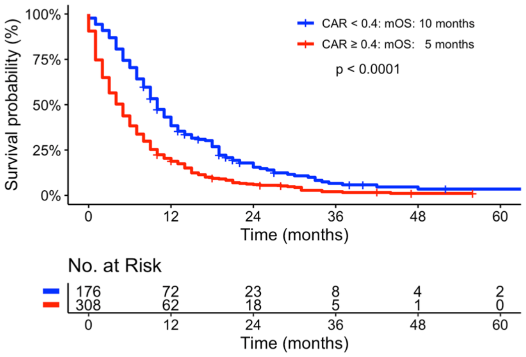

(c)

CAR - CRP to albumin ratio; CRP – C-reactive protein.

Figure S6. Kaplan-Meyer Curves univariate comparison of sub-group analyses for ECOG status 0-1 of: (**a**) NLR; (**b**) LMR; (**c**) PLR; (**d**) CAR; (e) IBI.

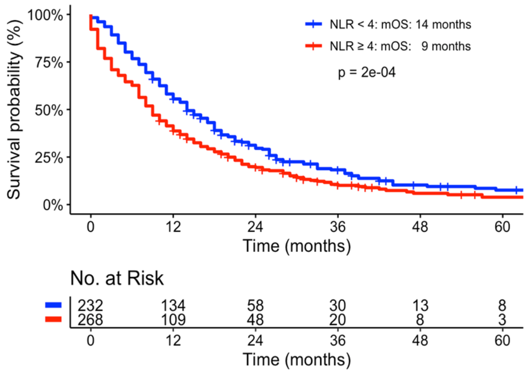

(a)

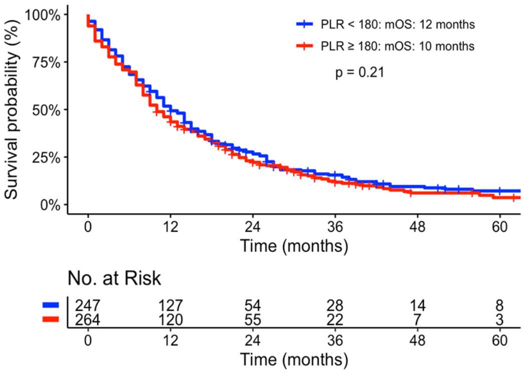

(b)

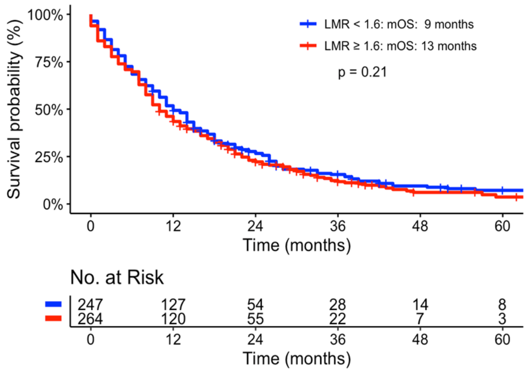

(c)

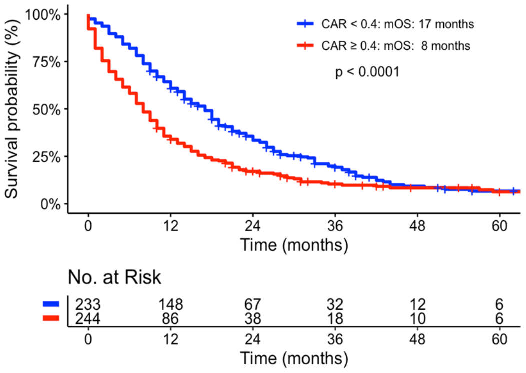

(d)

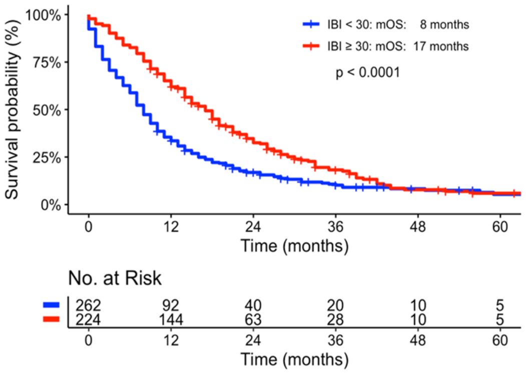

(e)

NLR - neutrophile to lymphocyte ratio; PLR - platelet to lymphocyte ratio; LMR - lymphocyte to monocyte ratio; CAR - CRP to albumin ratio; CRP – C-reactive protein; IBI – Inflammatory Benchmark-Index.

Figure S7. Kaplan-Meyer Curves univariate comparison of sub-group analyses for ECOG status >2 of: (**a**) NLR; (**b**) LMR; (**c**) PLR; (**d**) CAR; (e) IBI.

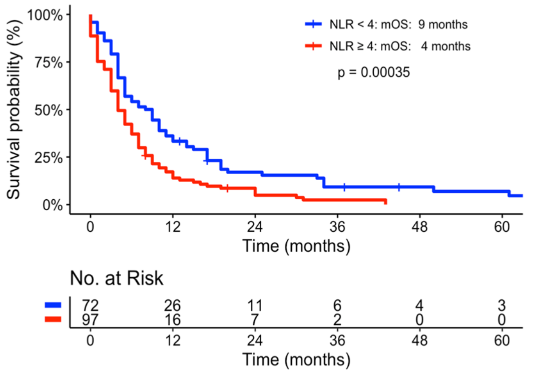

(a)

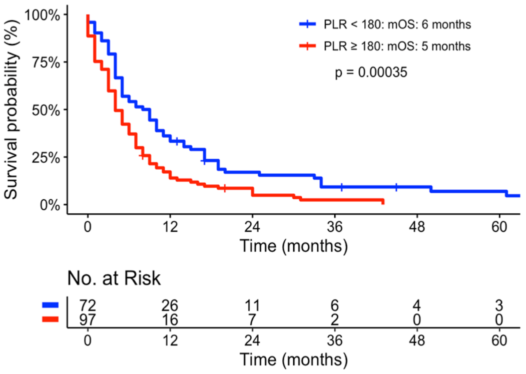

(b)

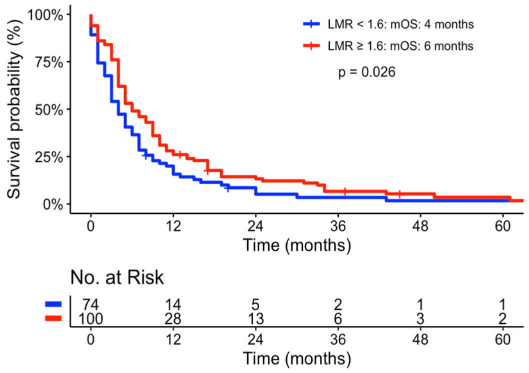

(c)

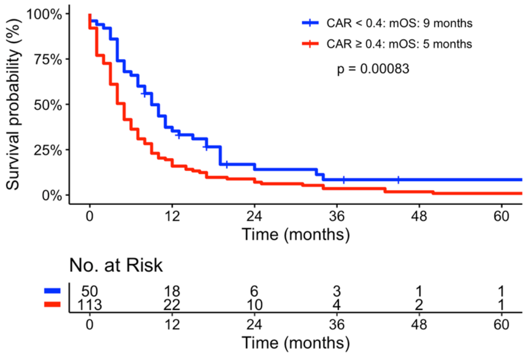

(d)

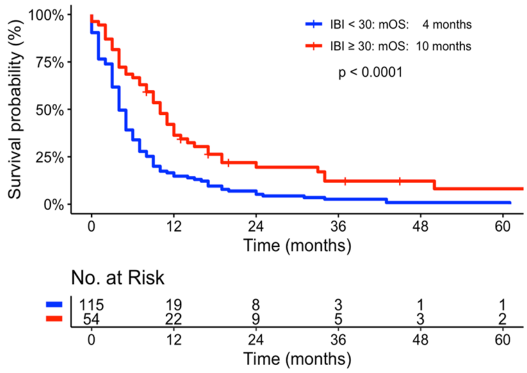

(e)

NLR - neutrophile to lymphocyte ratio; PLR - platelet to lymphocyte ratio; LMR - lymphocyte to monocyte ratio; CAR - CRP to albumin ratio; CRP – C-reactive protein; IBI – Inflammatory Benchmark-Index.
